# A single amino acid determines preference between phospholipids and reveals length restriction for activation ofthe S1P_4 _receptor

**DOI:** 10.1186/1471-2091-5-12

**Published:** 2004-08-06

**Authors:** Gill Holdsworth, Daniel A Osborne, TrucChi Thi Pham, James I Fells, Gillian Hutchinson, Graeme Milligan, Abby L Parrill

**Affiliations:** 1 Department of NCE Biology, Celltech R&D Ltd., 216 Bath Road, Slough, Berks., SL1 4EN, U.K; 2 Department of Chemistry and Computational Research on Materials Institute, The University of Memphis, Memphis, Tennessee 38152, USA; 3 Molecular Pharmacology Group, Division of Biochemistry and Molecular Biology, Institute of Biomedical and Life Sciences, University of Glasgow, Glasgow G12 8QQ, Scotland, UK

## Abstract

**Background:**

Sphingosine-1-phosphate and lysophosphatidic acid (LPA) are ligands for two related families of G protein-coupled receptors, the S1P and LPA receptors, respectively. The lysophospholipid ligands of these receptors are structurally similar, however recognition of these lipids by these receptors is highly selective. A single residue present within the third transmembrane domain (TM) of S1P receptors is thought to determine ligand selectivity; replacement of the naturally occurring glutamic acid with glutamine (present at this position in the LPA receptors) has previously been shown to be sufficient to change the specificity of S1P_1 _from S1P to 18:1 LPA.

**Results:**

We tested whether mutation of this "ligand selectivity" residue to glutamine could confer LPA-responsiveness to the related S1P receptor, S1P_4_. This mutation severely affected the response of S1P_4 _to S1P in a [^35^S]GTPγS binding assay, and imparted sensitivity to LPA species in the order 14:0 LPA > 16:0 LPA > 18:1 LPA. These results indicate a length restriction for activation of this receptor and demonstrate the utility of using LPA-responsive S1P receptor mutants to probe binding pocket length using readily available LPA species. Computational modelling of the interactions between these ligands and both wild type and mutant S1P_4 _receptors showed excellent agreement with experimental data, therefore confirming the fundamental role of this residue in ligand recognition by S1P receptors.

**Conclusions:**

Glutamic acid in the third transmembrane domain of the S1P receptors is a general selectivity switch regulating response to S1P over the closely related phospholipids, LPA. Mutation of this residue to glutamine confers LPA responsiveness with preference for short-chain species. The preference for short-chain LPA species indicates a length restriction different from the closely related S1P_1 _receptor.

## Background

Sphingosine-1-phosphate (S1P) and lysophosphatidic acid (LPA) are phospholipid growth factors which are present in normal serum and plasma. These lipids elicit diverse responses from a wide range of cell types, including enhanced cell survival, cell proliferation, induction of cytoskeletal changes and chemotaxis (reviewed in [1-4]. Some of these responses reflect activation of G protein-coupled receptors of the endothelial differentiation gene (Edg) family. The Edg receptor family is classified into two clusters based on ligand selectivity: S1P_1/2/3/4/5 _(formerly Edg1/5/3/6/8) specifically respond to S1P whilst LPA_1/2/3 _(formerly Edg2/4/7) respond to LPA [5]. Members of the S1P receptor family display higher sequence similarity to each other (approximately 40% identity) than to members of the LPA receptor family (approximately 30% identity). These homologies, coupled with observed differences in the structure of S1P and LPA receptor genes, suggest that these receptor families evolved from distinct ancestral genes. The S1P receptors contain a conserved glutamic acid residue present within the third TM that corresponds to glutamine in the LPA receptors. Interaction between distinct functional groups present on S1P and LPA with this residue was shown for the S1P_1 _and LPA_1 _receptors using computational modelling techniques [6,7] and was demonstrated as the basis for the ligand preference displayed by the receptors. Experimental characterisation confirmed that replacement of glutamic acid with glutamine in S1P_1 _changed ligand specificity from S1P to LPA, and the reciprocal mutation in LPA_1 _resulted in recognition of both LPA and S1P [7].

In the present study, the role of this residue in determining ligand selectivity for the S1P_4 _receptor was examined. Phylogenetic analysis of the Edg family of receptors indicates that S1P_4 _is more closely related to other S1P receptors than receptors which respond to LPA. However, S1P_4 _lies on the edge of the S1P family cluster and has been shown to bind S1P with lower affinity than other S1P receptors and hence it has been suggested that S1P is not the true endogenous agonist of this receptor [8]. We therefore decided to investigate whether replacement of this residue (E^3.29(122)^) with glutamine conferred LPA-responsiveness to the S1P_4 _receptor and hence determine the role of this residue in this lower-affinity S1P receptor. To achieve this, we expressed wild type and E^3.29(122)^Q mutant S1P_4 _receptors in CHO-K1 cells and studied responses to lysophospholipids using a [^35^S]GTPγS binding assay. Since CHO-K1 cells respond to LPA, we utilised fusion proteins constructed between the S1P_4 _receptor and a pertussis toxin-insensitive Gα_i1_(C^351^I) G protein. Expression of these proteins in CHO-K1 cells followed by treatment with pertussis toxin prior to harvest allowed elimination of any signal due to stimulation of endogenous LPA receptors. Within this study, we also examined how the length of the LPA acyl chain affected potency at the mutant S1P_4 _receptor, using a panel of naturally occurring LPA analogues. Computational models of complexes between the wild type or mutant S1P_4 _receptor and S1P and LPA species were used to provide a molecular interpretation of the experimental findings.

## Results

**Figure 1:**
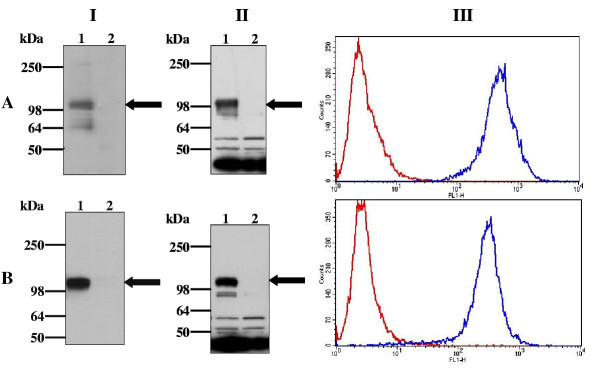
**Expression of HA-S1P_4_-Gα_i1 _and HA-S1P_4_(E^122^Q)-Gα_i1_(C^351^I) in CHO-K1 cells. **Membranes from untransfected CHO-K1 cells (lane 2) and CHO-K1 cells stably expressing HA-S1P_4_-Gα_i1_(C^351^I) (A) or HA-S1P_4_(E^122^Q)-Gα_i1_(C^351^I) (B) were analysed by Western blotting using anti-HA (panel I) or anti-Gα_i1 _(panel II) antibodies. Visualisation of immunoreactive proteins was achieved using chemiluminescence after incubation of the blot with appropriate HRP-conjugated secondary antibodies. The position of each HA-S1P_4 _fusion protein is indicated by an arrow. Cell-surface expressed HA-S1P_4 _receptor was detected by FACS analysis (panel III) using a Fluorescein conjugate of the anti-HA antibody (blue line). Cells were also stained with an isotype matched control antibody (red line). No staining of untransfected CHO-K1 cells was observed using the Fluorescein conjugate of the anti-HA antibody (not shown). Data are presented as overlay histograms and are representative of at least five independent experiments.

Human HA-S1P_4 _was mutated at position 122 to replace the naturally occurring glutamic acid with glutamine. The mutant and wild type receptors were stably expressed in CHO-K1 cells as in-frame GPCR-G protein fusions with pertussis toxin-insensitive Gα_i1_(C^351^I). Western blotting was used to detect expression of these fusion proteins. Membranes from HA-S1P_4_-Gα_i1_(C^351^I)- or HA-S1P_4_(E^3.29(122)^Q)-Gα_i1_(C^351^I)-transfected cells contained a polypeptide with an apparent molecular mass of approximately 110 kDa, which reacted with anti-HA and anti-SG1 antibodies (Figure 1A and 1B) and was consistent with expression of the GPCR-G protein fusion. Confirmation of comparable cell-surface expression of these proteins was obtained via FACS analysis using an anti-HA antibody directly conjugated with fluorescein (Figure 1 Panel III).

**Figure 2:**
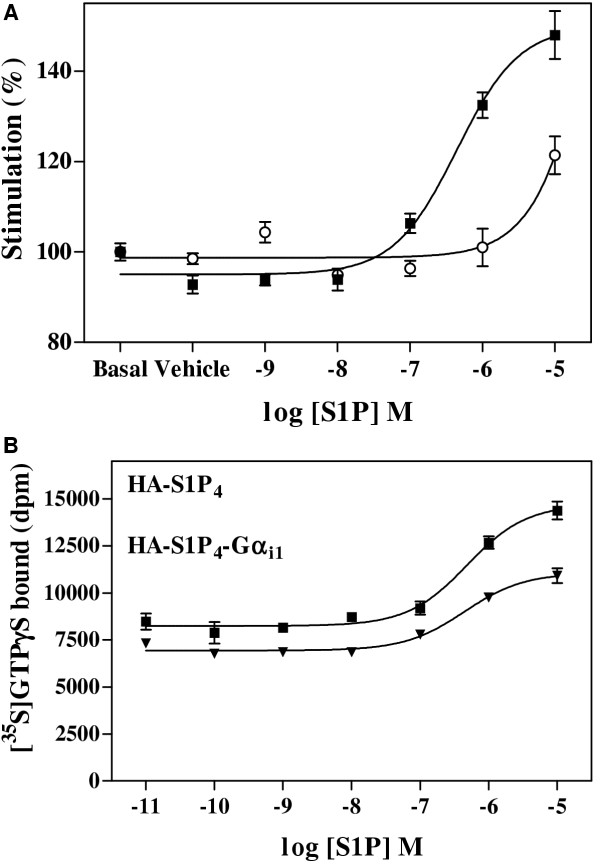
**Sensitivity of HA-S1P_4_-Gα_i1_(C^351^I) and HA-S1P_4_(E^3.29(122)^Q)-Gα_i1_(C^351^I) to S1P in [^35^S]GTPγS binding assay. **A. Membranes from CHO-K1 cells transfected with HA-S1P_4_-Gα_i1_(C^351^I) (*filled squares*) or HA-S1P_4_(E^3.29(122)^Q)-Gα_i1_(C^351^I) (*open circles*) which had been cultured in the presence of 100 ng/mL pertussis toxin for 24 hours prior to harvest were stimulated with varying concentrations of S1P for 30 minutes at 30°C in the [^35^S]GTPγS binding assay. Gα_i _G proteins were immunoprecipitated after solubilisation and preclearance with non-immune serum. Data are the mean of three determinations ± SEM from a single experiment, and are representative of three such experiments performed. B. Dose-dependent stimulation of wild-type HA-S1P_4 _(*filled squares*) and HA-S1P_4_-Gα_i1 _fusion protein (*filled triangles*) by S1P measured as described in panel A. These data are representative of three such experiments performed and analysis of mean EC_50 _values obtained for each protein showed them to be not statistically different.

The response of HA-S1P_4_-Gα_i1_(C^351^I) and HA-S1P_4_(E^3.29(122)^Q)-Gα_i1_(C^351^I) to S1P was assessed using membranes from cells transfected to express these proteins and treated with pertussis toxin prior to harvest. S1P promoted dose-dependent increase in [^35^S]GTPγS binding to membranes containing HA-S1P_4_-Gα_i1_(C^351^I) with an EC_50 _of 355 nM ± 155 nM (n = 3); in contrast, membranes from HA-S1P_4_(E^3.29(122)^Q)-Gα_i1_(C^351^I)-expressing cells demonstrated severely impaired response to S1P (Figure 2A). The EC_50 _for S1P stimulation of the HA-S1P_4_-Gα_i1 _fusion protein (355 ± 155 nM) was not statistically significantly different from that obtained using the unfused HA-S1P_4 _receptor (439 ± 187 nM, Figure 2B) and compared favourably with published values for this receptor in HEK293T cells from two different research groups of 270 nM [9] and 790 nM [10].

**Figure 3:**
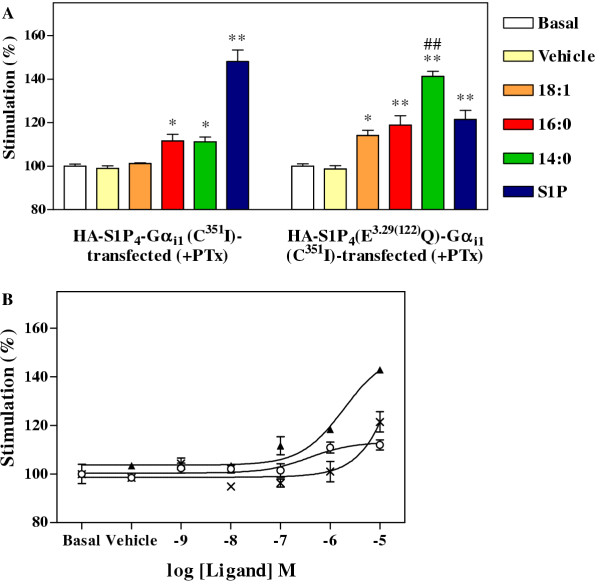
**Ligand preference of HA-S1P_4_(E^122^Q)-Gα_i1_(C^351^I) in [^35^S]GTPγS binding assay. **Membranes were stimulated for 30 minutes at 30°C with lysophospholipid ligands in the [^35^S]GTPγS binding assay and Gα_i _G proteins immunoprecipitated after solubilisation and preclearance with non-immune serum. (A) Membranes from CHO-K1 cells transfected to express HA-S1P_4_-Gα_i1_(C^351^I) or the mutant HA-S1P_4_(E^3.29(122)^Q)-Gα_i1_(C^351^I) and incubated with 100 ng/mL pertussis toxin for 24 hours prior to harvest, were left untreated (basal), or treated with vehicle or 10 μM concentrations of 18:1 LPA, 16:0 LPA, 14:0 LPA or S1P. Data are the mean of three determinations ± SEM from a single experiment and are representative of three such experiments performed. Statistical significance from the basal responses of each set of membranes tested is denoted by * (P < 0.05) or ** (P < 0.01); ## denotes statistical significance from the response to 18:1 LPA (P < 0.01) for the HA-S1P_4_(E^3.29(122)^Q)-Gα_i1_(C^351^I)-transfected membranes. (B) Membranes from CHO-K1 cells transfected to express HA-S1P_4_(E^122^Q)-Gα_i1_(C^351^I) and incubated with 100 ng/mL pertussis toxin for 24 hours prior to harvest, were stimulated with various concentrations of S1P (*crosses*), 18:1 LPA (*open circles*) or 14:0 LPA (*filled triangles*). Data are the mean of three determinations ± SEM and are representative of three such determinations performed.

Structure activity relationships were determined by the [^35^S]GTPγS assay for S1P_4 _or its E^3.29(122)^Q mutant using S1P and LPA species with 14:0, 16:0 and 18:1 acyl chains at a single (10 μM) concentration. Of the lysophospholipids tested, only S1P induced a strong response over basal levels (approximately 48% ± 5%) in membranes containing HA-S1P_4_-Gα_i1 _(Figure 3A), whilst 18:1 LPA did not stimulate a statistically significant response; weak stimulation of [^35^S]GTPγS binding was observed with 14:0 and 16:0 LPA (approximately 11% ± 3% above basal in each case). In contrast, membranes containing HA-S1P_4_(E^3.29(122)^Q)-Gα_i1_(C^351^I) showed at least weak response to each ligand (Figure 3A). The weakest agonist at the E^3.29(122)^Q mutant S1P_4 _receptor was 18:1 LPA, which produced only 14% ± 2% stimulation over basal. S1P and 16:0 LPA gave approximately 21% ± 4% and 19% ± 4% stimulation over basal response, respectively. The best agonist for the E^3.29(122)^Q mutant was 14:0 LPA, which gave a 40% ± 2% enhancement over basal levels. The stimulation promoted by 14:0 LPA was statistically different from that produced by 18:1 LPA (p < 0.01). Sensitivity of the receptor to stimulation by each form of LPA, and particularly 14:0 LPA, was markedly increased after introduction of the E^3.29(122)^Q mutation and indicated that this position was important in influencing HA-S1P_4 _ligand preference.

These results indicate that introduction of the E^3.29(122)^Q mutation in the S1P_4 _receptor confers LPA-responsiveness, and that a short form of LPA was a more effective agonist than the intermediate and longer forms, when tested at this single concentration. Dose response curves were constructed for ligand-induced activation of the E^3.29(122)^Q S1P_4 _mutant by the 14:0 and 18:1 forms of LPA as well as S1P (Figure 3B). An EC_50 _could only be determined for the 14:0 form of LPA as S1P and 18:1 LPA caused minimal stimulation at only the highest concentration tested. The EC_50 _value for activation of HA-S1P_4_(E^3.29(122)^Q)-Gα_i1_(C^351^I) was calculated to be 3.8 ± 1.4 μM. However, since a plateau of maximal stimulation was not achieved, interpretation of this EC_50 _value needs caution. This result clearly showed that 14:0 LPA was a weak agonist of HA-S1P_4_(E^3.29(122)^Q)-Gα_i1_(C^351^I) and hence confirmed the involvement of residue 122 in S1P_4 _ligand preference. Similar results were obtained using a second CHO-K1 clone expressing this fusion protein (not shown).

**Figure 4:**
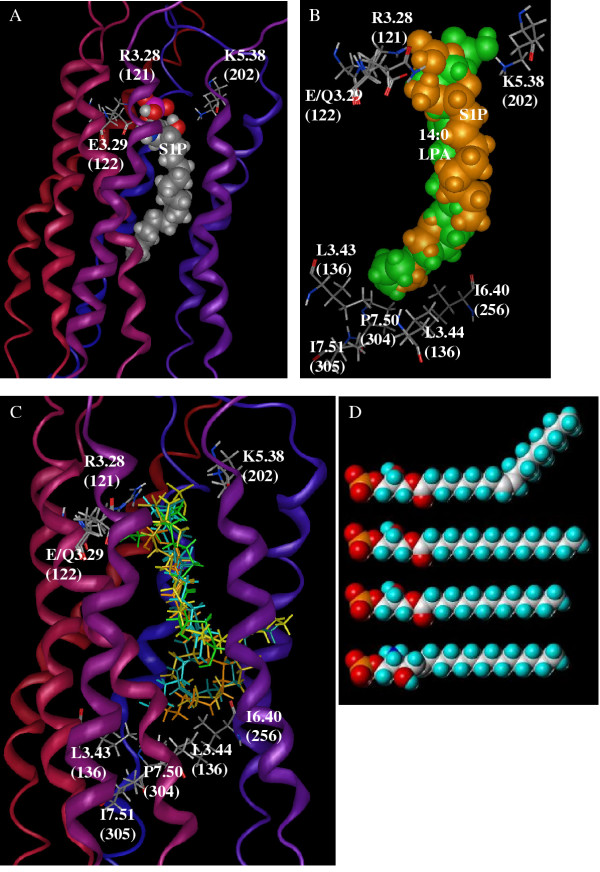
**Computational models of wild type S1P_4 _and its E3.29(122)Q mutant with S1P and LPA species. **Computational models of the complexes between the wild type S1P_4 _or its E^3.29(122)^Q mutant with S1P or various LPA species generated by Autodock 3.0 and minimised using the MMFF94 forcefield in the MOE program. Complexes in each panel are shown from the same viewpoint with the extracellular end of the receptors oriented to the top of the figure. Standard element color codes are used with grey, white red, blue and magenta representing carbon, hydrogen, oxygen, nitrogen and phosphorous. Ribbons are shaded from red at the amino-terminus to blue at the carboxy-terminus. (A) Model of the complex between S1P (spacefilling) and the wild type S1P_4 _receptor. Residues in the receptor involved in ion pairs with S1P are shown as stick models and labelled. (B) Superimposition of the wild type S1P_4 _complex with S1P (orange) and the E^3.29(122)^Q S1P_4 _mutant complex with 14:0 LPA (green). For clarity, the only position at which the modelled amino acid position is shown for both receptor models is 3.29(122). Other residues had very similar optimised positions in the two model structures. (C) Superimposition of wild type S1P_4 _complexes with 18:1 LPA (cyan), 16:0 LPA (yellow) and 14:0 LPA (green) on E^3.29(122)^Q mutant complexes with 18:1 LPA (blue-green), 16:0 LPA (gold) and S1P (orange). For clarity, the only position at which modelled amino acid position is shown for both the wild type and mutant receptor models is 3.29(122). Other residues had very similar optimised positions in all model structures. (D) Space-filling models which represent the minimised extended conformation of each structure were constructed using SYBYL 6.9 software (Tripos Inc., St. Louis, MO., U.S.A.). The distance between phosphorus and terminal carbon atoms was predicted for each structure listed from top to bottom: 18:1 LPA, 27.0 Å; 16:0 LPA, 26.7 Å; 14:0 LPA, 24.2 Å; S1P, 24.0 Å.

Computational modelling of the S1P complex with the wild type S1P_4 _receptor identifies the best S1P binding site within the TM with the phosphate group at the extracellular end (Figure 4A). Ion pairs appear between the phosphate group of S1P and two cationic amino acids, R^3.28(121) ^and K^5.38(202)^. An additional ion pair occurs between the cationic ammonium of S1P and E^3.29(122)^. Hydrophobic residues from TM2, TM3, TM5 and TM6 line the binding pocket and surround the alkyl chain of S1P.

The best complex of 14:0 LPA in the E^3.29(122)^Q S1P_4 _mutant receptor model has striking similarity to the best complex of S1P in the wild type S1P_4 _receptor model (Figure 4B). Both models demonstrate ion pairing between the phosphate group and two cationic amino acids, R^3.28(121) ^and K^5.38(202)^. Each ligand interacts with the amino acid at position 3.29(122), S1P by an ion pair with the carboxylate of the wild type glutamate and 14:0 LPA by a hydrogen bond with the mutated glutamine. Multiple hydrophobic residues surround the nonpolar tails of the lipid ligands. The superimposition of the two complexes (Figure 4B) also demonstrates that the ligands occupy almost identical volumes. Common interactions and overlap volumes are qualitatively consistent with the experimental findings that these ligands give similar 48% and 40% maximal stimulation over basal for S1P at the wild type and 14:0 LPA at the mutant receptor, respectively.

In contrast to the complexes of 14:0 LPA with E^3.29(122)^Q S1P_4 _and S1P with wild type S1P_4_, the remaining complexes show much less common volume (Figure 4C). Most complexes exhibit the phosphate interactions described for 14:0 LPA with E^3.29(122)^Q S1P_4 _and S1P with wild type S1P_4_. Of particular interest is the observation that the best complexes generated by Autodock for the 18:1 LPA species with wild type S1P_4 _has a very high positive van der Waals interaction energy, > 3000 kcal/mol, compared to values well under 200 kcal/mol for every other complex studied. In the best complexes found for 16:0 LPA and 18:1 LPA in both constructs, the terminal six to eight carbons of the hydrophobic tails fold into L-shaped conformations quite different from the extended conformations observed in the S1P complex with wild type S1P_4 _or the 14:0 LPA complex with the E^3.29(122)^Q S1P_4 _mutant. The terminal carbons in several complexes curl out of the receptor between TM5 and TM6 (Figure 4C) due to the restricted length of the binding pocket. These results suggest that the complete lack of S1P_4 _activation in response to 18:1 LPA is likely due to failure to form a complex. The strongest complexes formed, S1P with wild type S1P_4 _and 14:0 LPA with the E^3.29(122)^Q S1P_4 _mutant, have complementary interactions with the residue at position 3.29(122). These strong complexes give the most robust activation. Weak complexes are formed for other combinations due to mismatched interactions with position 3.29(122) or excessive length of the hydrophobic tail. The presence of hydrophobic tails of 16:0 or 18:1 LPA between transmembrane domains may additionally impair the conformational change necessary for full agonist responses.

## Discussion

Parental CHO-K1 cells respond to LPA in functional assays, reflecting expression of endogenous LPA_1 _(G. Holdsworth, *et al*., manuscript in preparation). For this reason, fusion proteins between wild type or mutant HA-S1P_4 _and the pertussis toxin-insensitive Gα_i1_(C^351^I) G protein were used in these studies. Expression of these proteins in CHO-K1 cells followed by treatment with pertussis toxin prior to harvest allowed elimination of any signal due to stimulation of endogenous LPA receptors. McAllister *et al*. [11] (.(adopted a similar approach for studies of the LPA_1 _receptor.

We examined the role of residue E^3.29(122) ^in controlling S1P_4 _ligand selectivity using functional and computational methods. This residue, which is conserved throughout the S1P receptors, has been shown to control ligand specificity for the related S1P_1 _receptor [7]. Introduction of the E^3.29(122)^Q mutation severely affected the response of S1P_4 _to S1P: in dose-response experiments S1P caused minimal stimulation at only the highest concentration of ligand used. This is in agreement with published observations for activation of the equivalent S1P_1 _mutant [7]. 14:0 LPA was able to induce dose-dependent stimulation of S1P_4_(E^3.29(122)^Q) with an EC_50 _of approximately 3.8 μM but only promoted minimal stimulation of the wild type S1P_4 _receptor. The modelled complexes of 14:0 LPA with E^3.29(122)^Q S1P_4 _and S1P with wild type S1P_4 _demonstrate nearly identical volumes occupied by the two ligands and very similar interactions between these ligands and their respective receptors. Of particular importance are amino acid residues at positions 3.28(121), 3.29(122) and 5.38(202), which either ion pair with the phosphate or interact with the 2-amino or 2-hydroxyl group in S1P and 14:0 LPA, respectively. The importance of interactions with amino acids at positions 3.28 and 3.29 has been previously noted for the S1P_1 _[6,7] and LPA_1,2,3 _[7,12] receptors.

**Figure 5:**
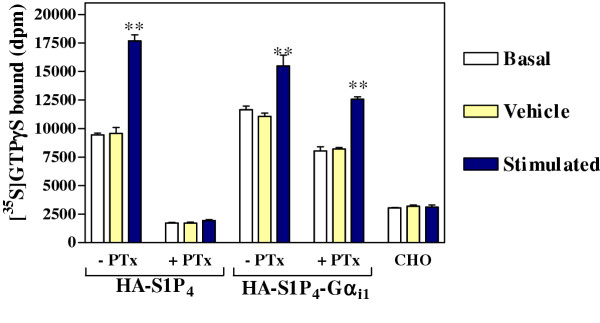
**Constitutive activity of HA-S1P_4 _and HA-S1P_4_-Gα_i1_(C^351^I). **Comparison of basal, vehicle and stimulated (10 μM S1P) GTPγS binding for membranes prepared from CHO cells transfected to express HA-S1P_4 _or HA-S1P_4 _fused to PTx insensitive Gα_i1_. Where indicated, cells were treated with 100ng/ml PTx for 24 hours prior to harvesting. PTx treatment of HA-S1P_4_-transfected membranes prevented activation by S1P and also caused a dramatic reduction in basal signalling, indicative of constitutive activity. In contrast, when the HA-S1P_4_-Gα_i1 _fusion-expressing membranes were treated with PTx, there was only a slight reduction in basal signalling and the receptor still responded to exogenous S1P, indicating that the receptor signalled via the tethered, PTx-insensitive G protein. Basal signalling in PTx-treated HA-S1P_4_-Gα_i1_-expressing membranes exceeded that seen with membranes from untransfected CHO cells.

The S1P_4 _receptor exhibits marked constitutive (agonist-independent) activity (Figure 5) which was unaffected by the introduction of the E^3.29(122)^Q mutation (data not shown). This indicates that the mutation perturbs S1P recognition without affecting the ability of the receptor to spontaneously adopt an active conformation. Similar observations have been reported for the β_2_AR, where a mutation in the sixth transmembrane domain abolished agonist activation but not constitutive activity [13].

Unlike S1P, which exists as a single species *in vivo*, the term LPA actually refers to a family of molecules that take the general form 1-*o*-acyl-2-hydroxy-*sn*-glyceryl-3-phosphate. Naturally occurring forms of LPA contain acyl chains of differing lengths, with differing degrees of saturation. Investigations into the effect of the length and degree of saturation of the acyl chain of LPA have been undertaken for the LPA receptors [14,15], but limited SAR information is available for S1P receptors (22). The LPA-responsive E^3.29(122)^Q S1P_4 _mutant facilitates structure activity relationship (SAR) studies due to the greater availability of LPA analogs relative to S1P analogs. Comparison of space-filling models of the structures of S1P and three analogues of LPA (Figure 4D) revealed that 14:0 LPA most closely resembled S1P in terms of apparent length. [^35^S]GTPγS binding assays demonstrated greater agonist activity of 14:0 LPA at the mutant receptor relative to 18:1 or 16:0 LPA. This SAR indicates a length restriction for the S1P_4 _agonist binding site. Model complexes of 16:0 and 18:1 LPA contained alkyl chains that fold at the bottom of the binding pocket, defined by a cluster of hydrophobic amino acids. Three of these differ either in position of sidechain branching or size relative to LPA receptors and the other S1P receptors. Position 2.46, I88 in S1P_4_, is leucine in LPA_1–3 _and other S1P receptors. Residue I6.40(256) is larger than the valine found in the other four S1P receptors, LPA_1 _and LPA_3_. Finally, I7.51(305) corresponds to the smaller valine in S1P_2 _and S1P_3 _and the much smaller alanine in LPA_2_. These findings provide a molecular explanation for a similar SAR observed using para-alkyl amide analogs of S1P [9]. SAR obtained with the S1P_4 _mutant are in contrast to that shown by LPA receptors, which exhibit the general trend of 18:1 ≥ 16:0 > 14:0 for potency and maximal stimulation [15].

Since mutation of residue 122 in the S1P_4 _receptor from the naturally occurring glutamic acid to glutamine conferred responsiveness to 14:0 LPA and severely affected responses to S1P, our observations support the hypothesis that this conserved residue in the third transmembrane domain of the S1P receptors is involved in ligand recognition. This is in contrast to a recent paper describing models of several GPCRs, including S1P_4_, which had been generated using novel first principle methods [16]. In this model of S1P_4_, interactions between S1P and residues T^7.34(127) ^and W^7.37(291) ^and E^7.30(284) ^were observed. Interaction of E^7.30(284) ^with the ammonium group of S1P appeared to control ligand selectivity since the other residues appeared to interact with the phosphate group, which is present on both LPA and S1P. It is therefore surprising that none of these residues are conserved throughout the S1P or LPA receptor families. The data presented here support the assertion that glutamic acid residue 3.29 present in the third transmembrane domain of the S1P receptors controls ligand selectivity and suggest that the S1P_4 _model described by Vaidehi *et al*. [16] is inaccurate.

The current study provides new information for the development of more selective S1P receptor agonists. In particular, an S1P analog with its hydrophobic chain extended by either 2 or 4 carbons would be a very poor agonist of the S1P_4 _receptor. On the other hand, the activation of the S1P_1_-E^3.29(121)^Q mutant by 18:1 LPA [7] indicates that a chain-extended S1P analog should retain agonist activity at the S1P_1 _receptor. S1P receptor agonists with differing selectivity profiles will be useful tools to more completely map the physiological and pathophysiological roles of these receptors.

## Conclusions

These studies confirm that glutamic acid residue 3.29, present in the third transmembrane domain of the S1P receptors is important for the selective recognition of S1P, versus the closely related lipid, LPA. Mutation of E3.29 to glutamine diminished response to S1P and allowed structure activity studies using the diverse available LPA species. The mutant S1P_4 _receptor is stimulated most strongly by LPA 14:0 and is not activated by the longer LPA 18:1, in contrast with a previous report on the analogous S1P_1 _receptor mutant that responded to LPA 18:1. Thus the S1P_4 _receptor ligand binding pocket is shorter in length than the S1P_1 _ligand binding pocket.

## Methods

### Residue nomenclature

Amino acids within the TM of S1P_4 _can be assigned index positions to facilitate comparison between receptors with different numbers of amino acids, as described by Weinstein and coworkers [17]. An index position is in the format x.xx. The first number denotes the TM in which the residue appears. The second number indicates the position of that residue relative to the most highly conserved residue in that TM which is arbitrarily assigned position 50. E3.29, then, indicates the relative position of glutamate 122 in TM3 relative to the highly conserved arginine 143 in the E(D)RY motif which is assigned index position 3.50 [17].

### Materials

Materials for tissue culture were supplied by Invitrogen Ltd. (Paisley, Scotland, U.K.). Foetal bovine serum was obtained from Helena Biosciences Ltd., (Sunderland, U.K.) or PAA Labs GmbH., (Linz, Austria). Pertussis toxin was purchased from CN Biosciences Ltd., (Nottingham, U.K.). Lysophosphatidic acid (18:1, 16:0 and 14:0) and S1P were from Avanti Polar Lipids Inc., (Alabaster, AL., U.S.A.). The SG1 antiserum was produced previously [18]. All other chemicals were from Sigma Aldrich Company Ltd., (Gillingham, Dorset, U.K.) or BDH Ltd., (Poole, Dorset, U.K.) unless stated otherwise.

### Construction of receptor expression plasmids

The S1P_4 _coding sequence was cloned from a human PBMC cDNA library using the sense primer 5'-GAGAGA**GCGGCCGC**CACCATGTATCCATATGATGTTCCAGATTATGCTAACGCCACGGGGACCCCGGTG-3', which contains a *Not*I restriction site (bold) and the haemagglutinin HA epitope tag (YPYDVPVYA, underlined) immediately after the initiator methionine, and the antisense primer 5'-GAGAGA**GAATTC**GGCGATGCTCCGCACGCTGGAGATG-3', which contains an *Eco*RI restriction site (bold) and changes the S1P_4 _stop codon to alanine (underlined). A C^351^I mutant of the Gα_i1 _G protein (previously produced, [19]) was amplified using PCR with the sense cloning primer 5'-GAGAGA**GAATTC**GCCA CCATGGGCTGCACACTGAGCG-3', which contains the *Eco*RI restriction site (bold), and the antisense cloning primer 5'-GAGAGA**GGATCC**TTAGAAGAGACCGATGTCTTTTA G-3', which contains a *Bam*HI restriction site (bold). After digestion of each PCR product with the appropriate restriction enzymes, fragments were ligated into the pIRESpuro mammalian expression vector (Invitrogen Ltd.) to generate an in-frame fusion between HA-S1P_4 _and Gα_i1_(C^351^I).

The E^3.29(122)^Q mutation was introduced into the S1P_4 _sequence in parallel PCR reactions. Complementary oligonucleotides were designed across the residue which was to be mutated such that each primer contained the necessary base change to mutate residue 122 to glutamine (underlined in each primer): sense mutational primer: 5'-CAGTGGTTCCTACGGCAGGGCCTGCTCTTCAC-3'; antisense mutational primer: 5'-GTGAAGAGCAGGCCCTGCCGTAGGAACCACTG-3'. Mutational sense or antisense primers were used in parallel PCR reactions with the appropriate antisense or sense cloning primer, with HA-S1P_4 _plasmid DNA as template. Equimolar amounts of each purified PCR product were mixed and amplified in a further reaction, using the cloning primers described above. The resultant product was digested with the appropriate restriction enzymes and ligated with the Gα_i1 _sequence in the pIRESpuro expression vector to generate an in-frame fusion between HA-S1P_4_(E^3.29(122)^Q) and Gα_i1_(C^351^I).

### Cell culture and transfection

CHO-K1 cells were maintained at 37°C with 5% CO_2 _in Dulbecco's modified Eagle's medium (DMEM), supplemented with 10% foetal bovine serum (FBS), 2 mM L-glutamine and non-essential amino acids. Sub-confluent cell monolayers were stably transfected to express either HA-S1P_4_-Gα_i1_(C^351^I) or HA-S1P_4_(E^3.29(122)^Q)-Gα_i1_(C^351^I) fusion proteins using Lipofectamine reagent (Invitrogen). 72 hours post-transfection, cells were seeded in media supplemented with 7.5 μg/mL puromycin and the resultant clones examined for expression of cell surface receptor using FACS analysis. Clonal cell lines were expanded in complete DMEM containing 7.5 μg/mL puromycin and were transferred to serum free DMEM approximately 24 hours prior to harvesting. Where indicated, 100 ng/mL pertussis toxin was included in the serum free medium.

It should be noted that we initially expressed S1P_4 _in RH7777 cells, which are unresponsive to S1P and LPA and have been commonly used for studies of Edg family receptors [20]. Unfortunately, our attempts to detect activation of S1P_4 _expressed in these cells using a variety of functional assays were unsuccessful. Therefore, we used CHO-K1 cells as an alternative host in these studies; expression of functional S1P_4 _in CHO-K1 cells has also been reported by Mandala et al. [21].

### FACS analysis

The amino-terminal HA-epitope tag was detected using a fluorescein conjugate of the anti-HA antibody, clone 3F10 (Roche Molecular Biochemicals Ltd., Lewes, U.K.). Cells were harvested non-enzymatically and washed with FACS buffer (PBS containing 3% FBS and 0.1% NaN_3_) then stained with the 3F10 antibody (or an isotype matched control) for 40 minutes at 4°C in the dark. After washing with FACS buffer, cells were analysed using a FACScalibur flow cytometer (BD Biosciences, Oxon., U.K.).

### Preparation of cell membranes

Cells were harvested non-enzymatically, washed with PBS and resuspended in "assay buffer" (20 mM Hepes, pH 7.4, 3 mM MgCl_2_, 100 mM NaCl), supplemented with "complete" protease inhibitors (Roche Molecular Biochemicals Ltd.). Cells were homogenised in a nitrogen cavitation chamber (500 psi for 15 minutes). Unbroken cells and nuclei were pelleted by centrifugation (500 × *g*, 10 minutes, 4°C) and the supernatant fraction was centrifuged at 45,000 × *g *for 45 minutes at 4°C. Membrane pellets were resuspended in assay buffer, titurated through a fine gauge needle and stored at -80°C until required.

### Immunoblot analysis

Samples were resolved by SDS-Page on 4–20% Tris-Glycine gels (Invitrogen) and were transferred to Immobilon-P membrane (Millipore Ltd., Herts., U.K.). The membrane was blocked using 2.5% Marvel in PBS before incubating with primary antibodies which had been diluted in PBS/0.1% Tween-20 containing 1% Marvel. The high affinity rat anti-HA antibody was diluted 1 in 500; the anti-G_αi1 _antibody (Autogen Bioclear Ltd., Wilts., U.K.) was diluted 1 in 1000. Immunoreactivity was detected using an appropriate horseradish peroxidase-conjugated secondary antibody, diluted 1 in 10,000 in PBS/0.1% Tween-20 containing 1% Marvel, followed by detection using SuperSignal reagents (Perbio Science Ltd., Cheshire, U.K.).

### [^35^S]GTPγS binding assay

[^35^S]GTPγS binding experiments were performed essentially as described previously [22]. Briefly, membranes were incubated with or without the indicated ligand for 30 minutes at 30°C in assay buffer containing [^35^S]GTPγS (100 nCi/point), saponin (20 μg/point) and 0.1 μM GDP. 18:1 LPA was prepared as a 2 mM DMSO stock whilst 16:0 and 14:0 LPA were prepared as 2 mM stock solutions in 1:1 ethanol:water per supplier recommendation due to their poor solubility in DMSO. S1P had previously been dispensed as thin film aliquots (dissolved in MeOH and the solvent evaporated under nitrogen) in brown glass vials and stored at -70°C prior to use. Lipids (S1P or LPA forms) were diluted in assay buffer containing 1% fatty acid free BSA, such that the final concentration of BSA in the assay was 0.1%. Following incubation, membrane protein was solubilised with 1.25% NP-40 and 0.4% SDS and after pre-clearance using non-immune serum, Gα_i1/2 _subunits were immunoprecipitated with SG1 antiserum, used at a dilution of 1 in 200. Non-specific binding was determined by the addition of 100 μM GTPγS. Bound radioactivity was measured using liquid scintillation counting.

### Experimental data analysis

Numerical data are expressed as means ± standard error, shown as error bars in the appropriate figures. Statistical comparisons were made using one-way ANOVA with Dunnett's multiple comparison post test.

### Receptor model development

A model of human S1P_4 _(GenBank™ accession number AAP84350) was developed by homology to the experimentally-validated model of S1P_1 _[23]. Alignment of the S1P receptor sequences was performed using the MOE software package (version 2003. 01 ed. Chemical Computing Group, Montreal, Canada). The alignment was optimised by the manual removal of gaps within the TM, and alignment in the region of TM5 was shifted one position to correctly orient K5.38(202) toward the interior of the helical bundle (Pham, *et al*., unpublished data). A preliminary model was generated by homology modelling using default parameters and subsequently manually refined to optimise interhelical hydrogen bonding. Cis-amide bonds present in the loop regions were converted to the trans conformation by manual rotation followed by the minimisation of two residues on either side of the amide linkage to a root mean square (RMS) gradient of 0.1 kcal/mol·Å using the MMFF94 forcefield [24]. After these manual refinements, the receptor model was optimised using the MMFF94 forcefield to an RMS gradient of 0.1 kcal/mol·Å.

A model of S1P_4 _with the E^3.29(122)^Q mutation was developed by performing the appropriate mutation in MOE, and saturating the residue with hydrogen atoms. To allow the sidechains of the other residues in the binding pocket to adapt to the presence of the new moiety, the backbone atoms of the receptor were fixed and the receptor was optimised to an RMS gradient of 0.1 kcal/mol·Å using the MMFF94 forcefield [24].

### Ligand model development

Computational models of the naturally-occurring stereoisomers of 14:0 LPA, 16:0 LPA, 18:1 LPA, and S1P were built using the MOE software package. The -1 ionization state for the phosphate functionality was chosen for all ligands, and the +1 ionization state was chosen for the amine moiety of S1P. Previous docking studies using the -2 ionization state for phosphate in related systems yield essentially identical geometries as studies using the -1 ionization state. These ligands were geometry optimised using the MMFF94 force field [24].

### Docking

Using the AUTODOCK 3.0 software package [25], 14:0 LPA, 16:0 LPA, 18:1 LPA, and S1P were docked into the S1P_4 _wild type and S1P_4 _E^3.29(122)^Q mutant receptor models. Each docking box was centered near F^3.33(126) ^with dimensions of 30.75 × 23.25 × 23.25 or 32.25 × 23.25 × 23.25 Å for shorter (S1P and 14:0 LPA) or longer (16:0 and 18:1 LPA) ligands, respectively. At least 20 putative complexes were generated for each receptor:ligand pair using docking parameters at default values with the exception of the number of energy evaluations (2.5 × 10^8^), generations (10000) and maximum iterations (3000). Resultant complexes were evaluated based on final docked energy, Van der Waals interaction energies from the MMFF94 forcefield as well as visual analysis. The complexes with the lowest final docked energies and others of interest were geometry optimised using the MMFF94 force field [24], and the lowest energy complex after minimisation was chosen as the final complex structure.

## Abbreviations

CHO, Chinese hamster ovary; Edg, endothelial differentiation gene; ERK, extracellularly regulated kinase; FACS, fluorescence activated cell sorter; G protein, guanine nucleotide-binding protein; GPCR, G protein-coupled receptor; HA, haemagglutinin; LPA, lysophosphatidic acid; MAP, mitogen-activated protein kinase; PBMC, peripheral blood mononuclear cell; PTx, pertussis toxin; SIP, sphingosine-1-phosphate; TM, transmembrane domain

## Authors' contributions

G Holdsworth performed and interpreted all studies with experimental S1P_4 _fusion proteins and drafted the manuscript. D Osborne performed and interpreted docking studies to generate all mutant complexes with all LPA species and S1P and all wild type complexes with LPA species. TC Pham generated the homology model of the human S1P_4 _receptor. J Fells performed docking studies of S1P with the wild type S1P_4 _receptor. G Hutchinson and G Milligan participated in the design and coordination of the experimental studies with S1P_4 _fusion proteins. A Parrill participated in the design and coordination of the modelling studies and edited the manuscript.
